# Therapeutics

**Published:** 1878-12

**Authors:** 


### THERAPEUTICS.

THERAPEUTICS OF DIPHTHERIA DURING THE YEAR 1877.—Ferrand. *L'Union Médicale*. July 9, 1878, page 37.

"The treatment of diphtheria is a question always open and always present, and unfortunately the constant progression of this terrible malady, of which the frequency and gravity appear to increase from year to year, imposes upon every physician the duty of devoting his attention to it." (Cadet de Gassicourt, in *Bull. de Thérap.*) Readers of the *Union Médicale*, in which the statistics are published every three months, by M. Besnier, may judge that these words are not exaggerated; they will then justify the care taken to observe the progress and the acquisitions of therapeutics in a matter where it may render such great services.

In presence of a disease in which the product possesses in a high degree, specific characters, it would seem that the search after a specific remedy should be permitted, and should be encouraged. Without wishing to introduce here this grave question of morbid specificity, we may be permitted, nevertheless, to observe, that there are diseases more specific still than diphtheria, less obscure in their causes, more constant in their effects, and in face of which one should give up the search after a specific remedy, and strive on the contrary, to progress in the rational search after agents capable of modifying the seat or physiological form of this disease.

The current which at present draws us toward anti-septics, is felt especially in the therapeutics of diphtheria. In presence of a disease of which the principal objective character consists in an excrementitial production, so to speak, which deposits itself upon certain surfaces, spreads after the manner of a parasite, decomposes to a putrid magma, very certainly capable of a septic resorption, nothing is more natural than to think of employing anti-septics.

The good effects which have resulted from the atomization of carbolic acid in catarrhal affections of the respiratory organs (see my last review, *The Therapeutics of Phthisis in* 1877), should encourage the employment of such a method in the treatment of diphtheria.

With the same end in view, Dr. Créquy has proposed to the Therapeutic Society, not only the application of tannin in powder or collyrium, as Trousseau did, but also a fumigation by means of boiling water in which tannin has been deposited. It is known that the vapor of water carries with it a notable portion of tannin in its natural state.

The salicylate of sodium, tried internally by M. Cadet de Gassicourt (loc. cit.), has given results of but slight importance. Salicylic acid had already succeeded no better in the hands of M. Bergron.

In another communication to the Therapeutic Society Dr. Soulez has extolled carbolized camphor, which is nothing but a solution of camphor and carbolic acid in alcohol: 25 grams of camphor in powder dissolved in a mixture of 1 gram of alcohol and 9 of carbolic acid, constituting this medicament, which is used pure, or mixed with oil of almonds. On touching frequently with this mixture the false membrane of croup, it is soon seen to wither and disappear, leaving a simple ulceration, which heals rapidly. It will be seen that it is not solely an antiseptic M. Soulez has hit upon. At any rate, the four happy examples which he cites to support his theory encourage further trials of his remedy. It is not surprising that, antiseptic and balsamic at the same time, carbolized camphor should arrest the alteration of the morbid product once formed, and modify advantageously the secretions of the mucous membrane upon which it is produced.

It is in this way, indeed, that the most significant effects have been proved, and it is by means of chlorate of potassium that they have been especially obtained. Since the remarkable labors of our regretted colleague, Isambert, chlorate of potassium has been constantly used in the treatment of angina and croup. Dr. Seligmuller praises it anew, and insists upon the efficaciousness of solutions of this salt. He recommends giving this solution free from all mixture, every hour at least, and considers all other means as capable of some moral effect, useful for the friends, but without real benefit to the patient. Cessation of fetidity of the mouth, disappearance gradually of the false membranes, reparation of the ulcerations, and finally rapid amelioration of the general condition, these are the effects which Seligmuller attributes to the internal use of chlorate of potassium.

Guided by the favorable local action of chlorate of potassium, Isambert considered it as a very useful element in the medication of diphtheria. Our author makes almost a specific of it. Without going so far as that, it may be admitted that chlorate of potassium has only a direct local action on the false membranes; its passage after absorption into the salivary glands and into the pharyngeal mucous membrane, which appear to be its place of elimination, certainly explain in great part its utility in the diverse anginas, and in diphtheritic angina. This utility is a fact which I have proven many times. I believe less in a favorable action of chloride of potassium upon the nutrition and general state of the subjects. I have often observed that the stomach tolerated it with difficulty, and I have encountered patients who refused to take it. Seligmuller himself recommends looking after the heart and digestive functions, which this agent may injure. But these reservations well known, I think it one of the most precious agents for combatting a malady which resists so many others: it acts as a modifier of the secretions of the mucous membrane diseased, and as an antiseptic on the products secreted by this membrane. As to its general action, and the oxygen which it brings into the blood, deprived of this gas by the bacteria of diphtheria, it is a fact which remains to be demonstrated.

Furthermore, the divers communications which have been made to the Therapeutic Society agree in praising the use of chlorate of potassium in the treatment of diphtheria. Bucquoy R. Blache, and myself, have insisted upon its good effects. In the comparative study which he has made in his service at St. Eugénie, on the use of chlorate of potassium, cubebs, and salicylate of sodium, in the treatment of diphtheria, Cadet de Gassicourt does not hesitate to accord the first place to chlorate of potassium, which he regards as much superior to the others, without giving it heroically or as a specific. In a word, if there is any specific action, it appears to be only local, from direct action at the moment of ingestion, or secondary, at the moment of elimination. It is a species of therapeutic specificity which perhaps does not merit the name, but which we have already distinguished from a true specific action.

Dr. Trideau has returned, in the *Gazette Hebdom.*, to the utility which there is in the use of cubebs alone, or with copaiba, in diphtheria. These agents, it is true, are not easily taken; but well masked in a potion like the following: 15 grams of powder of cubebs, 50 grams of syrup, and 50 grams of Malaga wine, or as dragées, etc., they may serve as excellent adjuncts to the treatment, always difficult, and in which the physician must often use as much firmness as knowledge. The action of the balsams upon the respiratory mucous membrane is too evident to refuse putting them to use in these cases; but it would be a grave mistake to use them as specifics.

I might say as much of the tincture of eucalyptus, which Dr. Walcher has used and recommended (*Gaz. Méd. de Strasbourg*). After having prescribed an ipecac, he gives a syrup composed of 10 grams of the alcoholate of eucalyptus in 38 grams of simple syrup. This dose may even be exceeded in the twenty-four hours. No doubt the very exciting action of eucalyptus may modify advantageously the general state of the patient, at the same time that its toxic action, direct and by elimination, bears especially upon the diseased points.

All these agents, it may be seen, are drawn from the domain of the modifiers of the secreting surface and of its products of secretion; it is a local alterant action, or an antiseptic influence which is especially sought in them. For a long time we have remarked into what discredit the caustics properly so called have fallen. Nevertheless, the elder Guillon does not appear to share this opinion; for, since 1828 he has not ceased to treat diphtheritic pharyngitis by insufflations of powdered nitrate of silver. (*Gaz. méd. de l'Algérie.*) This physician recommends even to extend the insufflation not only to the diphtheritic surfaces, but also above and below them, and by that he thinks to prevent extension. I can scarcely share his confidence. Having seen many times with what facility diphtheria spreads to the mucous and even cutaneous surfaces, when they are the seat of any inflammatory irritation, and above all when they begin to ulcerate, I always feared that the action of the caustic, whether superficial or destructive, might offer to the diphtheria new surfaces which it is already too much disposed to invade, and to open with them new doors to septic or specific resorption. Therefore, for a long time I have employed as a toxic a collyrium made with powder of cinchona, as I have seen my preceptor, Dr. Delpech, do. With Rose Cormack elsewhere (*Edinburgh Med. Journ.*), I believe the most inoffensive toxics are the best; and I limit myself by preference to those which he advises ; a mixture of glycerine and borax, or an acidulous solution (hydrochloric acid), or alkaline (lime water), or neuter (sulphate of sodium), all very dilute. I believe even that the passage from one to the other of these means is often useful, in face of an inflamed mucous membrane, of which the vascularization modifies itself better under the alternate action of divers toxics, than by an action always the same, whatever its efficacy.

CONCERNING THE ADMINISTRATION OF QUININE. (*The Doctor, Sept.* 1, 1878.)

According to Mr. Batterbury one grain of quin. sulph. dissolved in an ounce of milk hardly renders it bitter; two or three grains only slightly so. Five grains may be taken in a tumblerfull without any unpleasant sensations.

Mr. Collier proposes the use of the kinate of quinia for hypodermic use, this salt being soluble in three or four parts of water. He prepares a basic kinate from the calcic kinate, and adds to it, in solution, the quinia sulphate in powder, procuring the amorphous kinate by evaporation. This is subsequently readily prepared for use. It possesses the two great attributes of solubility and neutrality.

M. Yvon recommends the lactate of quinia for use in the same manner, while the sulphorinate is also stated to be suitable for the same purpose.

With reference to the administration of the drug to nursing women, M. Burdel finds that the drug is absorbed more rapidly when given on an empty stomach, less so when given with the food—i. e., it appears in the mother's milk in larger or smaller proportion, according to the time it was ingested. So that when it becomes necessary to administer quinine soon after delivery, its injurious effects may be avoided by giving it with meals, or with some food, and by emptying the mother's breast with the pump three hours after the dose.

Infants at the breast never suffer from malaria, no matter how cachectic the mother, until after the fourth month; this immunity may last to the period of dentition.

Codeia in Cancer of the Pylorus. (*New Remedies.*)

Professor Austin Flint recommends 0.06 of codeia most highly, in cancers of the outlet of the stomach, saying that in such a case it completely stopped the emesis and pain, and the patient even thought the tumor was decreasing in size.

Heinsch's Sugar Test for Dangerous Organic Matter in Water.

Place a quantity of the water in a clean, glass stoppered bottle; add a few grains of pure sugar, and expose to the light in a window of a warm room. If the water becomes turbid, even after exposure for a week, reject it; if it remains clear it is safe.

Ammonio-Sulphate of Copper in Epileptiform Facial Neuralgia.

Frère, of the Lariboisière, has found this, in doses of 0.07 twice a day, succeed well, when other means had failed. In one case 0.10 were given at one dose.

Warts.

Dr. Craig, of Montreal, recommends a four per cent. solution of chloral hydrate, as a painless but effectual application to warts.

